# How to Organise the Country

**Published:** 1919-12-13

**Authors:** 


### THE THREE LEAN HOSPITAL YEARS AHEAD.

### HOW TO ORGANISE THE COUNTRY.

EVERY British man and woman, aye, and the children too, who salute each other on New Year's Day, 1920, should thank God that at last we have a woman M.P. in the House of Commons. The first woman M.P., Lady Astor, in the few days of her membership of the House, has aroused new ideas and awakened ambitions in the hearts of men and women to cease to be humbugs and to shed the unrealities which mark much of the procedure followed by individuals enjoying the good things of this life in large or reasonable measure because they are hampered by procedure and follow the crowd. One favourite tradition with many people is to bother the sick and the hospitals, for whom and which money is always being asked, that they are tired of hearing the cry, and they make a virtue, or some of them do, of never giving anything to Christ's poor, even when sick, so as to discourage the annoyance of a request for money for a hospital or kindred institution. Those there are, many of them, less wealthy, who affirm that it is ruinous to attain notoriety by giving a large sum to a hospital because you can never escape the beggars afterwards, however many years you may linger on this earth. Others, again, and this is a surprising curse which very many wealthy people bear throughout their lives, suffer from the inability to make up their minds to give largely to anything in their lifetime; but the best of them intend in their hearts to leave something handsome in their wills. We have known a person of great wealth and great heart, too, who, when he learned to give largely to hospitals himself, was persuaded by the example and teachings of the apostle of personal service in the days of health in the cause of the sick, to devote time and energy to bringing to the notice of his individual friends, with money, the splendid opportunity of doing good which a gift to a named object would produce. He worked zealously among his friends, and he had many, for some three months. He drove home his own experiences and the joy money had brought to him, really for the first time in his life, since he had learnt to give wisely and largely to noble enterprises. As a result he reported to his friends that although in several cases he had succeeded in eliciting a promise of a substantial gift from man or woman who was fired by his purposeful energy as an advocate, not one single cheque was drawn, but in every case came the confession, "I must defer my gifts for inclusion in my will."

What has this to do with the woman M.P., Lady Astor, whose spirited words of wisdom, and her example, are calculated materially to aid the standard of living and conduct among all classes throughout Great Britain? Just this. At an informal Board meeting at the Children's Hospital, Great Ormond Street, where the Hospital City idea, fully expounded and advocated in the eleventh edition of the *Encyclopædia Britannica*, and often in the columns of THE HOSPITAL (though it is limited to a Children's Hospital City in this case), Lady Astor rightly and properly maintained that the people could carry out the scheme well enough if they made up their minds to save the enormous sums now spent in London and other big towns on sheer luxuries. This message should be spread by the Press throughout Great Britain, and driven home by hospital authorities, that it may enter the minds and hearts of nine-tenths of the inhabitants of every county, city, town, village, and hamlet throughout the land? Why? Because hospitals are a necessity to every inhabitant throughout the United Kingdom, and few of us could remain in health, and even live in any proper meaning of the word, without the aid which they bring to every man, woman, and child in the land when sick or injured.

It is not, as Lady Astor so forcibly contended, a question of charity at all. Charity applied to hospital gifts is a misnomer. Such gifts, rightly understood and deliberately given, as they should be in every case by rich and poor alike, are the best of all insurances open to the provider of the money in question, whether the sum be large or small, according to the means with which each individual is blessed. We can say, as the experience of a long life, that the men, the women, and the children who once realise the truth, in regard to help of any kind given by grateful hearts to hospitals and other agencies for the relief of the sick, are never likely to discontinue the practice, because, like the millionaire whose experience we have reproduced, they gain unconsciously, but in a remarkable and most helpful manner, the sense that

**The Three Lean Hospital Years Ahead**—(*continued*).
they are doing something with their lives that is worth doing, something which brings them joy and strength and the consciousness of realisation, that, the giving of oneself, *con amore*, to others less fortunate, makes the world and our daily life brighter and better, more uplifting and joyous than all else besides.

Before we attempt to apply the teachings of what we have here written we must borrow one experience. During five years of her war work Lady Astor came across a number of heroes, and not one of them wanted anything except that his children should get a decent start in life. That was not what they got now owing to what was called civilisation. She entertained advanced ideas about what the community or the State owed to its children—that is, children of all ages—in this connection. If the average citizen properly realised the conditions under which so many children were now born and brought up, there would be no difficulty about what was called social reform. It was only by people doing what they ought to do that England could be made merry. At present they simply gave what they thought they could afford to give, but she wanted them, and so do we, to give what they could not afford. Legislation would do nothing unless the people, as men and women, did what was decent and clean and right towards each other. "Let everybody look into his or her own heart and see if they did this. People were appalling humbugs. Let them cease to be humbugs." What wants doing at the moment is for the people in every community, large or small, throughout Great Britain to be brought to remember, that, the hospitals, everywhere throughout the land, have before them as an after-war legacy three lean years. These three lean years should be anticipated and provided for by free gifts, raised and banked by June 30, 1920. The brains of every man, woman and child amongst us should be quickened by the recognition, that gifts, to each of these sustentation funds for the voluntary hospitals, are really the most grateful and best memorial, to the men who have made the supreme sacrifice, or been maimed in the war. The sooner every county, city, town, and hamlet has this brought home to them, the better it will be for every one of us resident in Great Britain, and the more welcome, in the future, will the memory of this act be, to the individual giver.

Let us look at the facts as they are to-day. Throughout Great Britain is there a single exception to the fact, that, in every city or town some nine-tenths of the whole population give nothing whatever, to a hospital or to the aid of the injured and the sick? This is true not of one city but of all cities, not of one town but of all towns and counties, as has been shown by the careful investigations of disinterested public men and citizens, whose figures are demonstrably accurate, and the result here stated convincingly shown. How can this state of affairs be remedied with a minimum of difficulty and the maximum of speed? Lady Astor properly made a point of the feeling of independence which all our brave warriors have exhibited and rightly feel. We have amongst us in every centre of population, large or small, numbers of warriors who have been demobilised, brave fellows to whom we owe our liberty and life under Providence to-day, in these islands, who have seen and known, and many of them have experienced, the magnificence of the service rendered to the sick and injured through the voluntary hospitals of this country, compared with every other type of hospital known to civilised man. At the moment our glorious privilege is to demonstrate to the world that we are neither fools nor ingrates, much less hypocrites, and that we do realise as a people our indebtedness to our brave warriors of all the Services, naval and military, to our doctors, nurses, and the armies of noble women, V.A.D.s, and those of the numerous other women's services who did such splendid work throughout the war, and are every one of them the better for the experience, and the sense that they, like the men, have made good in the war and done their duty with the best, for the Homeland and its people of all classes. These warriors and women workers have demonstrated their love for their home and country to which they have returned or are now returning. They can testify with convincing, nay unanswerable, force to the facts we have advanced in regard to the dependence of every man, woman and child on the hospitals, directly and indirectly. They, we are confident, would welcome the opportunity to attend committees or take part in public meetings where the truth was told in regard to the hospitals' work, their pressing needs, their opportunity for further assistance to all types of people, and the claims which they have upon every healthy person, rich or poor, to give with full hearts, collectively, a peace offering which would represent a sum, adequate to carry all the hospitals in a given district, safely through the lean years ahead. Thus would be secured to the residents in their immediate neighbourhood, better and ampler hospital accommodation and protection, than they have ever been able to supply in the past.

Here, then, it will be seen, every centre, large or small, which has the blessings of hospital provision in its midst, has at hand an army of virile, brave, patriotic, convinced, experienced opinion, which, if organised and brought to bear upon the

**The Three Lean Hospital Years Ahead**—(*continued*).

inhabitants of every hospital centre, would speedily wipe out the disgrace, which at present attaches to any such centre, where it can be truthfully said, that, only one in ten of the inhabitants has ever contributed anything towards the provision of hospital care, trained and efficient nursing, the succour and upbringing of the children on lines, which will make them healthy, joyous, and industrious citizens of the best.

It is no use playing with the emergency of the moment, which is grave and pressing. The old lines, the continual milking of the same cow, and the unenthusiastic endeavour to secure from them, somehow, enough money to keep the hospitals open must be ended. What is wanted is a virile, alive, earnest, purposeful, convinced army of new workers, who will speedily find new givers. The latter will then be roused as men and women asleep, until they recognise, and are grateful for the recognition, that on them rests a clear privilege, the fulfilment of which will yield them in the end greater satisfaction, than anything they may have done in the whole of their past lives. Once organise the machinery on the right lines, and by Midsummer Day, 1920, with God's blessing, the financial crisis of our hospitals everywhere will be solved, and the time for an awakened people, inhabiting the true Merrie England which we all desire, will have come.

That the forecast just given is not a mere chimera, but a practical, demonstrable reality is shown, by the movements which have taken place throughout England since we commenced to explain the Hospital Crisis in these columns. Every day brings evidence of results. Liverpool led the van by its Lord Mayor calling a meeting and asking the citizens to provide £80,000 to carry the Liverpool hospitals over the three lean years ahead. That appeal has gone home, and one single donation amounted to no less than £20,000. At Manchester £20,000 has been given by one donor spontaneously, but the Lord Mayor has yet to follow Liverpool's lead and secure for the hospitals the needed provision for the three lean years. The London hospitals, as represented by a first gift to Guy's Hospital, whose splendid service was demonstrated forcibly in our issue of November 29, have already received their gift of £20,000. H.R.H. the Prince of Wales, whose marvellous energy is a splendid inspiration to every man and woman, and especially to the younger members of the race, presided at a dinner in aid of the Middlesex Hospital on Tuesday night, at which no less a sum than £52,773 was received. The women set an example to the whole of the women of the United Kingdom by showing what they could do. Princess Alice handed in a cheque for £5,073, collected at the Woman's Dinner, and "Mrs. Gossip," of the *Daily Sketch*, handed in £7,500, the result of a Shilling Fund. The Prince of Wales, who made a splendid chairman, declared, "The welfare of our hospitals is a question which has appealed to all of us, the subject seems to be so full of human interest." H.R.H. set an example to every chairman who may follow him throughout the land, by adding, "I feel encouraged to try to say what I feel about it in my own way." He declared "The Hospital is a centre combining the greatest skill in medicine and surgery, where any man, woman, and child can go—not necessarily the poverty-stricken—and can be, and are, treated for ills which are often beyond the power of the ordinary general practitioner. The work of the hospital is not charity; it is humanity. It is humanity which bids us help our fellows in distress. Rich and poor alike benefit from the results of experiments at the voluntary hospitals, and they benefit also from the experience of the men who are trained there and whom those hospitals send out to the community." The Prince of Wales asked all to give; those who had given already he asked to give again, and to realise that one cannot give too much. "Each should give for the sake of the nation and for the sake of the Empire. We must have healthy men and women to keep it strong and keep it free."

Finally we would impress upon all hospital managers throughout the land that the country is awake to the fact that the hospitals have Three Lean Years Ahead. It is not only the larger cities, of which we have given examples, that have already given proof of this. We have had to-day as we write two notable instances of the smaller ones. Warrington Hospital, with 118 beds, started a special appeal and speedily raised £10,000 for past deficiencies and to provide a little surplus. Mansfield, a working-class district, with 100 beds, needs £5,000 to pay off an overdraft. This sum it is hoped to raise by a Shilling Fund and house-to-house collection on Christmas Day. It is further proposed to obtain the necessary money to increase the income by larger contributions from the work-people in the district covered by the hospital. We could multiply these examples, for instances are reaching us every day, that the movement, towards the Safety Fund, is now gaining force as the days pass. All that is wanted is courage, energy, conviction, and the co-operation, with renewed strength, of those who have made good in the war and are now home. All such will be found to have thankful hearts, willing to back the hospitals with their wonderful experiences, and to relieve those hospitals of their own district, from the present pressure of deficient means, to efficient and greater results.

